# Dual Sensory Impairment and Depressive Symptoms: Association and Pathways in Three Cohorts and a Meta‐Analysis

**DOI:** 10.1155/da/8488263

**Published:** 2026-09-29

**Authors:** Ping Ni, Li Liu, Li Zeng, Meiling Ge, Fang Wang, Xiuying Hu

**Affiliations:** ^1^ Innovation Center of Nursing Research and Nursing Key Laboratory of Sichuan Province West China Hospital Sichuan University/West China School of Nursing Sichuan University Chengdu China scu.edu.cn; ^2^ The Center of Gerontology and Geriatrics (National Clinical Research Center for Geriatrics) West China Hospital Sichuan University Chengdu China scu.edu.cn

**Keywords:** cognitive function, depression, hearing impairment, social engagement, vision impairment

## Abstract

**Background:**

The overall impact of dual sensory impairment (DSI) on depressive symptoms and its underlying mechanisms remains unclear, particularly across different culture contexts. This study aimed to examine the association between DSI and depressive symptoms in three distinct populations while exploring the mediating roles of social engagement and cognitive function.

**Methods:**

Participants were recruited from three aging cohorts: the China Health and Retirement Longitudinal Study (CHARLS), the Health and Retirement Study (HRS), and the English Longitudinal Study of Ageing (ELSA). Harmonized survey wave data (2011/2012, 2015/2016, and 2018) were analyzed. Depressive symptoms were assessed using the Center for Epidemiologic Studies Depression Scale (CES‐D), and DSI was defined based on self‐reported hearing and vision impairments. To address potential confounding, logistic regression models were adjusted for demographic, lifestyle, and health factors. Mediation analyses were conducted using the PROCESS Macro (model 4) to examine the roles of social engagement and cognitive function. Furthermore, a meta‐analysis was performed by integrating the results of the present study with those from previous cohort studies.

**Results:**

The study included 19,310 participants from the three cohorts. Separate analyses showed that DSI was significantly associated with higher odds of depressive symptoms across these cohorts (odds ratio [OR] = 1.24, 95% confidence interval [CI] = 1.15–1.33 in CHARLS; OR = 2.04, 95% CI = 1.78–2.31 in HRS; OR = 2.40, 95% CI = 1.79–3.00 in ELSA). A meta‐analysis confirmed this association (OR = 1.73 and HR = 1.30). This relationship was partially mediated by reduced social engagement (2.31%–6.35%) and cognitive decline (5.72%–14.00%).

**Conclusions:**

DSI is consistently associated with depressive symptoms in middle‐aged and older adults across China, the United States, and England. Both reduced social engagement and cognitive decline significantly mediated this association, with comparable effect sizes. The mediation effects are largely robust, with instability observed only in one sensitivity analysis.

## 1. Introduction

The accelerating pace of global population aging has brought significant challenges to public health systems [1]. Among these, depression stands as a critical concern, affecting over 280 million people worldwide [2], with 35.1% of older adults experiencing depressive symptoms [3]. As a leading cause of disability [4], depression contributes significantly to the global burden of disease [5]. In response, the World Psychiatric Association Commission has advocated for a prevention science perspective that considers risk and protective factors in preventing depression [6]. Sensory impairment, a common condition in older adults, is associated with poorer mental health and a greater risk of neuropsychiatric disorders [7, 8]; thus, it may represent a significant risk factor for depressive symptoms.

Dual sensory impairment (DSI), referring to the co‐occurrence of hearing and vision impairments, is common among older adults. Although numerous studies have demonstrated associations between individual sensory impairments and depressive symptoms, research on DSI remains limited. The relationship between DSI and depressive symptoms in middle‐aged and older adults remains unclear, with conflicting findings across studies [9–11] and a lack of meta‐analyses to synthesize the evidence. In addition, the underlying mechanisms are not well understood. Preliminary evidence suggests that DSI may lead to reduced social engagement and cognitive decline [7, 12, 13], which in turn could exacerbate depressive symptoms [14, 15]. Therefore, examining the role of social engagement and cognitive function in the DSI–depressive symptom link may help clarify potential mechanisms.

To address these gaps, this study examined the association between DSI and depressive symptoms using data from three large prospective cohorts and a meta‐analysis. We also explored whether social engagement and cognitive function mediate this association. Overall, our study provides evidence on this association across diverse populations through its multinational design and unified methodology and offers insights for developing interventions to reduce depressive symptoms in middle‐aged and older adults with DSI.

## 2. Methods

### 2.1. Study Design and Participants

This study used data from three prospective cohort studies: the China Health and Retirement Longitudinal Study (CHARLS), the Health and Retirement Study (HRS), and the English Longitudinal Study of Ageing (ELSA). These studies share similar designs and objectives. CHARLS recruited Chinese residents aged 45 years or older, while HRS and ELSA enrolled participants aged 50 years or older from the United States and England, respectively. Ethical approval was obtained from the Ethics Committee of Peking University (00001052‐11, 014), the Health Sciences and Behavioral Sciences Institutional Review Board at the University of Michigan (HUM00061128), and the National Research Ethics Service (MREC/01/2/91). Detailed information about each study is available elsewhere [16–18]. Informed consent was obtained from all participants. This study followed the Strengthening the Reporting of Observational Studies in Epidemiology (STROBE) reporting guidelines [19].

To ensure comparability across studies, we selected data from similar collection periods. The baseline assessments (CHARLS 2011; HRS and ELSA 2012) were followed by intermediate and final follow‐ups, with data collection extending to 2018 (Figure S1). Individuals were excluded if they were under 50 years of age, had missing data on the DSI or depressive symptoms, or presented with depressive symptoms at baseline. Ultimately, 5076 (CHARLS), 9163 (HRS), and 5071 (ELSA) individuals were included. Enrollment flow diagrams for all three cohorts are shown in Figures S2–S4.

### 2.2. Assessment of DSI

Sensory impairment was measured based on self‐reported hearing and vision function. In all three studies, hearing was evaluated with the question: “Is your hearing excellent, very good, good, fair, or poor (using a hearing aid as usual)?” Vision was assessed using two questions: (1) “How good is your eyesight for seeing things at a distance, like recognizing a friend from across the street (with glasses or corrective lenses if you wear them)?” and (2) “How good is your eyesight for seeing things up close, like reading ordinary newspaper print (with glasses or corrective lenses if you wear them)?” For all items, responses were recorded on a five‐point scale: (1) excellent, (2) very good, (3) good, (4) fair, and (5) poor. Sensory impairment was defined as a response of “fair” or “poor.” DSI was defined as the presence of both hearing and vision impairment [20].

### 2.3. Assessment of Depressive Symptoms

Depressive symptoms were assessed using the Center for Epidemiologic Studies Depression Scale (CES‐D). CHARLS used the 10‐item CES‐D (score range: 0–30), with higher scores indicating more severe depressive symptoms. Participants scoring ≥10 were classified as having depressive symptoms [21]. HRS and ELSA used the 8‐item CES‐D (score range: 0–8), with depressive symptoms defined as a score of ≥3 [22].

### 2.4. Assessment of Social Engagement

Social engagement was assessed through self‐reported participation in cultural, civic, and leisure activities over the past month [23]. CHARLS included 12 items, yielding a total score ranging from 0 to 33 [24]. HRS included 21 items, with a total score range from 21 to 147 [25]. ELSA included 11 items, with a total score ranging from 3 to 26 [23]. In all three studies, higher scores indicated greater social engagement. Details of the social engagement questions used in each study are provided in Table S1.

### 2.5. Assessment of Cognitive Function

Cognitive function was assessed across three domains: memory, orientation, and executive function. Memory was tested using a 10‐word list. The participants recalled the words immediately and after a delay. One point was given for each correct word. The memory score was the sum of both recall trials (range 0–20). Orientation was tested by asking for the current year, month, date of the month, and day of the week. One point was given for each correct answer (range 0–4). The executive function was tested using the serial sevens test. The participants subtracted 7 from 100 five times in a row. One point was given for each correct subtraction (range 0–5). A global cognitive function score was calculated as the sum of the three domain scores (range 0–29), with higher scores indicating better cognitive function. The reliability and validity of these tests are well‐established [26].

### 2.6. Covariates

The following three categories of covariates were selected based on previous studies [27, 28]. Demographic factors included age, gender, education, marital status, residence (CHARLS), and ethnicity (HRS and ELSA). Lifestyle factors included smoking and drinking status. Health status included the number of chronic diseases and activities of daily living (ADL). A high level of education was defined as having completed at least senior high school (12 years of education) [29]. Residence and ethnicity each indicate the structural social position in their respective cohorts. We therefore included both as proxies to control for confounding across cohorts. Chronic disease status was determined by self‐reported physician diagnoses (Table S2). ADL was assessed based on the Katz scale [30], and ADL disability was defined as having difficulty with at least one of the six basic daily activities [31] (Table S3).

### 2.7. Statistical Analysis

Baseline characteristics were presented as mean ± standard deviation (SD), median (interquartile range), or number (percentage).

For the primary analysis, incident depressive symptoms at the 2018 wave were treated as a binary outcome. Multivariable logistic regression was used to estimate the association between baseline DSI and incident depressive symptoms, and a random‐effects meta‐analysis was used to estimate the pooled odds ratio (OR) and 95% confidence interval (CI). Covariates were adjusted in three models: model 1 adjusted for age, gender, education, marital status, and residence/ethnicity; model 2 further adjusted for smoking and drinking; model 3 additionally adjusted for chronic disease and ADL. The maximum proportion of missing covariates was 5.6%. Missing data were handled using multiple imputation, with 20 imputed datasets generated under the missing‐at‐random assumption.

For the mediation analyses, depressive symptoms were treated as a continuous variable because this approach retains the full spectrum of symptom severity and avoids information loss due to dichotomization [32]. Mediation analysis was conducted in three steps: First, Pearson correlation analysis was used to assess associations among DSI, social engagement, cognitive function, and depressive symptom scores. Second, we employed PROCESS Macro (model 4) to analyze the mediation effects [33], with DSI as the independent variable, social engagement, and cognitive function as the mediators, with depressive symptom scores as the dependent variable, adjusted for all covariates. Third, the significance of mediation effects was evaluated using bootstrap with 5000 resamples; mediation was considered significant if the 95% CI did not include zero.

Sensitivity analyses were performed to test the robustness of the findings by (1) excluding participants with missing variables, (2) excluding those with baseline ADL disability, (3) excluding individuals with baseline emotional/nervous/psychiatric/memory‐related conditions, and (4) analyzing education as a multicategory variable in the CHARLS dataset. All covariate adjustments in the sensitivity analyses were consistent with those in the primary models.

All analyses were conducted using Stata Version 15.0 (StataCorp, College Station, TX) and SPSS Version 26.0 (IBM Corp., Armonk, NY, USA), with two‐tailed *p* < 0.05 considered statistically significant.

### 2.8. Meta‐Analysis of DSI and Depressive Symptoms

We conducted a meta‐analysis by combining results from the current study with those from previously published cohort studies that examined the association between DSI and depressive symptoms. The study protocol was registered with PROSPERO (CRD420251083208).

A systematic search was conducted across PubMed, Embase, Web of Science, and Medline from inception to 29 June 2025. Detailed search strategies are provided in Table S4. After removing duplicates with EndNote X9 (Clarivate Analytics, Philadelphia, PA, USA), two reviewers independently screened the titles/abstracts and then full texts for inclusion. Additionally, the reference lists of included studies and relevant reviews were manually searched for additional records. Studies were included if they met the following criteria: (1) prospective cohort design; (2) participants aged 45 years or older (refined from the PROSPERO protocol to harmonize cohort age ranges); (3) evaluated the association between DSI and depressive symptoms; (4) ORs or HRs and 95 % CIs were provided; (5) potential confounders were adjusted; and (6) published in English or Chinese. Extracted data included author, year, country, study population, sample size, age, proportion of women, follow‐up time, DSI, depressive symptom assessment methods, covariates, and risk estimates with 95% CIs in the maximally adjusted model. The Newcastle–Ottawa Scale (NOS) was used to assess the risk of bias [34]. Two reviewers (Ping Ni and Li Liu) independently conducted the literature screening, data extraction, and quality assessment. Any disagreements were resolved through discussion.

Heterogeneity was assessed using the *Q* statistic and *I* ^2^. Because ORs and HRs are not directly comparable, they were pooled separately using random‐effects generic inverse variance models to generate a pooled OR and a pooled HR. Although the original protocol specified a pooled RR, separate pooling was used to avoid combining nonequivalent measures. Sensitivity analyses were conducted to evaluate the robustness of the findings. Subgroup analyses were performed based on region, sample size, follow‐up duration, and the NOS score. Due to the small number of included studies, the originally planned Egger test and meta‐regression were not conducted [35].

All statistical analyses were performed using Stata Version 15.0 (StataCorp, College Station, TX). Two‐tailed *p* < 0.05 was considered statistically significant.

## 3. Results

### 3.1. Participant Characteristics

As shown in Table 1, the mean ages of participants in CHARLS, HRS, and ELSA were 60.2, 65.6, and 65.4 years, respectively. CHARLS included 54.3% of women, compared to 57.7% in HRS and 54.1% in ELSA. CHARLS also reported the highest prevalence of smoking (34.4%) and DSI (44.5%). HRS had the highest proportions of highly educated participants (82.2%) and those with multimorbidity (54.9%). ELSA had the highest prevalence of drinking (69.6%) and ADL disability (9.9%).

**Table 1:** Baseline characteristics of the study participants.

Characteristics^a^	CHARLS (*n* = 5076)	HRS (*n* = 9163)	ELSA (*n* = 5071)
Age (year)	60.2 ± 6.9	65.6 ± 9.3	65.4 ± 8.3
Female	2756 (54.3)	5285 (57.7)	2741 (54.1)
Rural resident	3077 (60.6)	—	—
White	—	6742 (73.6)	4943 (97.3)
High level of education	664 (13.1)	7529 (82.2)	3856 (76.0)
Married	4406 (86.8)	5900 (64.4)	3649 (72.0)
Smoking	1748 (34.4)	1041(11.4)	468 (9.2)
Drinking	1692 (33.3)	3908 (42.7)	3532 (69.6)
No. of chronic diseases			
0	1811 (35.7)	1646 (18.0)	1008 (19.9)
1	1595 (31.4)	2484 (27.1)	1391(27.4)
≥2	1671 (32.9)	5033 (54.9)	2672(52.7)
ADL disability	446 (8.8)	788 (8.6)	503 (9.9)
DSI	2257 (44.5)	539 (5.9)	122 (2.4)
Depressive symptoms	4 (2, 7)	0 (0, 1)	0 (0,1)

Abbrevations: ADL, activity of daily living; CHARLS, China Health and Retirement Longitudinal Study; DSI, dual sensory impairment; ELSA, English Longitudinal Study of Ageing; HRS, Health and Retirement Study.

^a^Continuous variables are reported as mean ± standard deviation (SD) or median (interquartile range). Categorical variables are reported as number (percentage).

### 3.2. Association Between DSI and Depressive Symptoms

At follow‐up, depressive symptoms were present in 1366 participants (26.9%) in CHARLS, 1121 participants (12.2%) in HRS, and 622 participants (12.3%) in ELSA. Overall, the DSI was significantly associated with higher odds of depressive symptoms (OR = 1.68, 95% CI = 1.43–1.94). Substantial heterogeneity was observed across the studies, with the strongest effect observed in ELSA (Figure 1).

**Figure 1:**
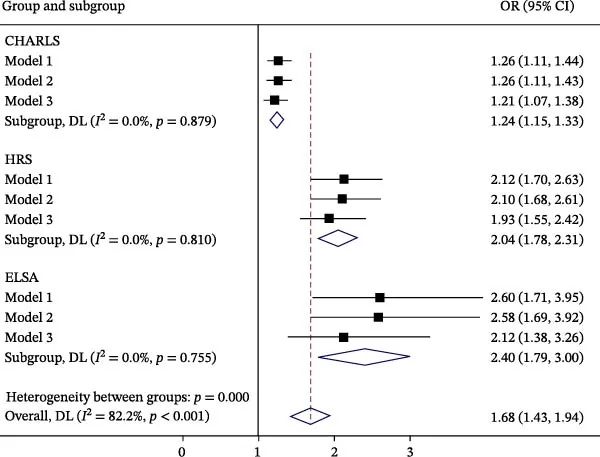
Pooled estimates from the three cohorts on dual sensory impairment and depressive symptoms.

### 3.3. Correlation Analysis

The results of correlation analysis were consistent across the three studies. DSI was negatively correlated with social engagement and cognitive function while it was positively correlated with depressive symptoms. Social engagement was negatively correlated with depressive symptoms. Cognitive function also showed a negative correlation with depressive symptoms (all *p* < 0.05) (Table S5).

### 3.4. Mediating Effect Analysis

In CHARLS, mediation analysis showed that DSI was a significant predictor of social engagement (*β* = −0.17, *p* = 0.0108), cognitive function (*β* = −0.47, *p* = 0.0001), and depressive symptoms (*β* = 0.36, *p* = 0.0219). Both social engagement (*β* = −0.14, *p* < 0.001) and cognitive function (*β* = −0.13, *p* < 0.001) were significant predictors of depressive symptoms (Figure 2a).

**Figure 2:**
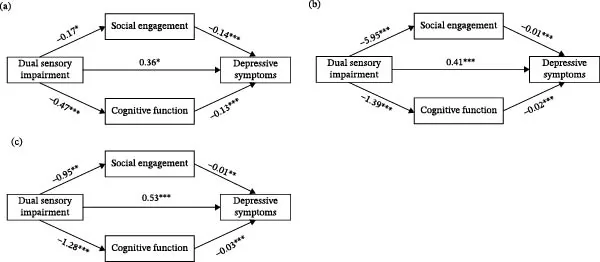
Mediation roles of social engagement and cognitive function in the association between dual sensory impairment and depressive symptoms (a) in the China Health and Retirement Longitudinal Study, (b) in the Health and Retirement Study, and (c) in the English Longitudinal Study of Ageing.  ^∗^ *p* < 0.05,  ^∗∗^ *p* < 0.01, and  ^∗∗∗^ *p* < 0.001.

In the HRS, mediation analysis showed that DSI was a significant predictor of social engagement (*β* = −5.94, *p* < 0.001), cognitive function (*β* = −1.39, *p* < 0.001), and depressive symptoms (*β* = 0.41, *p* < 0.001). Both social engagement (*β* = −0.01, *p* < 0.001) and cognitive function (*β* = −0.02, *p* < 0.001) were significant predictors of depressive symptoms (Figure 2b).

In ELSA, DSI was a significant predictor of social engagement (*β* = −0.95, *p* = 0.0052), cognitive function (*β* = −1.28, *p* = 0.0001), and depressive symptoms (*β* = 0.53, *p* < 0.001). Both social engagement (*β* = −0.01, *p* = 0.008) and cognitive function (*β* = −0.03, *p* < 0.001) were significantly associated with depressive symptoms (Figure 2c).

To assess the significance of the mediation effects, 95% CIs were estimated using the percentile bootstrap method with 5000 resamples. Notably, this estimate applies to symptom severity and cannot be directly compared with the ORs obtained from the logistic regression models. In all three cohorts, both social engagement and cognitive function significantly mediated the association, with comparable effect sizes (CHARLS: 5.33% and 14.00%; HRS: 6.35% and 6.32%; and ELSA: 2.31% and 5.72%) (Table 2).

**Table 2:** Bootstrap analysis of the mediating model.

Population	Path	Effect size	SE	95% CI		Mediated proportion (%)
				Lower	Upper	
CHARLS	Total effect	0.4465	0.1581	0.1366	0.7564	—
	Direct effect	0.3602	0.1571	0.0522	0.6682	80.67
	Total indirect effect	0.0863	0.0223	0.0446	0.1319	19.33
	Ind1: X→M1→Y	0.0238	0.011	0.0049	0.0472	5.33
	Ind2: X→M2→Y	0.0625	0.0187	0.0285	0.102	14.00
	Ind1 − ind2	−0.039	0.0211	−0.0824	0.0015	—
HRS	Total effect	0.4649	0.0662	0.3351	0.5947	—
	Direct effect	0.406	0.0665	0.2756	0.5364	87.33
	Total indirect effect	0.0589	0.01	0.04	0.0796	12.67
	Ind1: X→M1→Y	0.0295	0.0075	0.0154	0.0446	6.35
	Ind2: X→M2→Y	0.0294	0.0071	0.0163	0.0442	6.32
	Ind1 − ind2	0.0002	0.0107	−0.0209	0.0209	—
ELSA	Total effect	0.5804	0.1268	0.3319	0.8289	—
	Direct effect	0.5338	0.1266	0.2857	0.7819	91.97
	Total indirect effect	0.0466	0.0135	0.0227	0.0758	8.03
	Ind1: X→M1→Y	0.0134	0.0068	0.0023	0.0286	2.31
	Ind2: X→M2→Y	0.0332	0.0118	0.0135	0.0591	5.72
	Ind1 − ind2	−0.0198	0.0136	−0.0482	0.0055	—

*Note*: X, dual sensory impairment; M1, social engagement; M2, cognitive function; Y, depressive symptoms.

Abbrevations: CHARLS, China Health and Retirement Longitudinal Study; CI, confidence interval; ELSA, English Longitudinal Study of Ageing; HRS, Health and Retirement Study; SE, standard error.

### 3.5. Sensitivity Analyses

In sensitivity analyses, most results were consistent with the primary findings. However, in sensitivity analysis 1, the mediating effects of social engagement in CHARLS and cognitive function in HRS were no longer statistically significant (Tables S6–S25).

### 3.6. Meta‐Analysis

A total of 4175 studies were screened, and nine studies [9–11, 36–40] (including the current one) were included in the analysis (Figure S5). The characteristics of the included studies are presented in Table S26. Participants were from China, the United States, and Europe, with sample sizes ranging from 2703 to 56847. The included studies scored between seven and nine points, indicating a low risk of bias (Table S27).

The meta‐analysis demonstrated that DSI was significantly associated with depressive symptoms (OR = 1.73, 95% CI = 1.41–2.11; HR = 1.30, 95% CI = 1.19–1.42) (Figure 3). Sensitivity analyses showed that excluding any single study did not significantly change the pooled estimate (Figure S6). In the pooled HR analysis, region and NOS score were significant contributors to heterogeneity (both *p* = 0.043), whereas no significant sources of heterogeneity were identified in the pooled OR analysis (Table S28).

**Figure 3:**
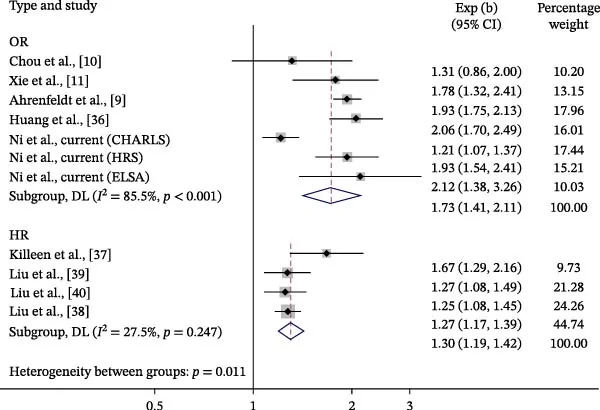
Meta‐analysis of cohort studies on dual sensory impairment and depressive symptoms.

## 4. Discussion

Based on three prospective cohort studies, this study examined the association between DSI and depressive symptoms and the mediating roles of social engagement and cognitive function. Overall, DSI was significantly associated with depressive symptoms in middle‐aged and older adults, a finding further confirmed by meta‐analysis. In the primary analyses, both reduced social engagement and cognitive decline significantly mediated this association, with comparable effect sizes. However, these mediation effects were not robust in sensitivity analysis 1. These results enhance our understanding of the pathways linking DSI to depressive symptoms in middle‐aged and older adults across countries and underscore the importance of addressing DSI to maintain mental health.

By integrating data from three national cohorts, this study consistently found that DSI was associated with depressive symptoms among middle‐aged and older adults in all three countries. This result aligns with findings from a European cohort study, which reported that DSI increased the risk of depressive symptoms by 0.93 times, with similar associations observed across European regions [9]. Another study found that neither hearing nor vision impairment alone was significantly associated with depressive symptoms, whereas DSI showed a clear association, highlighting the greater impact of dual impairments in later life [41]. Older adults with DSI are unable to use preserved visual or auditory function to compensate for a single sensory loss [42], and their daily functioning is more severely affected compared to those with a single impairment. These individuals face multiple stressors, including healthcare burdens, anxiety about treatments (e.g., intravitreal injections), fear of future sensory loss, and uncertainty about prognosis, all of which may contribute to depressive symptoms [43].

Mediation analyses indicated that DSI was associated with reduced social engagement and cognitive decline. Both mediators significantly mediated the association between DSI and depressive symptoms across all cohorts, with comparable effect sizes, although these effects were not robust in sensitivity analysis 1. Previous studies have reported similar associations between DSI and reduced social engagement in older adults [44]. After developing DSI, many older adults perceive their health more negatively [45] and show objective declines in functional ability [46], which may limit social activities and increase the risk of social isolation and depressive symptoms. DSI is also associated with cognitive decline [47]. Several hypotheses have been proposed to explain this connection, including the sensory deprivation hypothesis, the information degradation hypothesis, and the cognitive conformity hypothesis [48, 49]. Furthermore, cognitive decline has been well documented as a risk factor for depressive symptoms [15, 50, 51], supporting the mediating role of cognitive function in the DSI–depressive symptoms pathway.

In sensitivity analysis 1 (complete case analysis), the mediating effects of social engagement in CHARLS and cognitive function in HRS were no longer statistically significant. This may be explained by the reduced sample size and potential selection bias introduced by complete case analysis. These findings indicate that the mediation estimates are sensitive to the handling of missing data and should be interpreted with caution.

The heterogeneity observed in the meta‐analysis warrants emphasis. In the HR analysis, both region and NOS score were significant sources of heterogeneity. Chinese studies yielded a smaller pooled HR than studies from other regions. Subgroup analysis by the NOS score showed that high‐quality studies produced a smaller and more homogeneous estimate, whereas lower‐quality studies produced a larger and more heterogeneous estimate. A similar trend was observed in OR analysis, although it did not reach statistical significance. These findings suggest that region and study quality may influence both the magnitude and stability of the association. Given the substantial heterogeneity, the pooled OR and HR should be interpreted cautiously rather than as a single common effect across settings.

This study used data from multiple cohort studies to examine the association between DSI and depressive symptoms, explore potential mediating mechanisms, and guide cross‐national public health strategies. However, several limitations should be considered when interpreting the findings. First, DSI was based on self‐reported hearing and vision without objective verification. The binary definition did not capture the severity spectrum of impairments, a limitation shared with many previous studies that have adopted this approach for practical and comparability reasons [20, 52]. Second, cross‐cohort measurement heterogeneities should be considered when interpreting the findings. DSI prevalence varied substantially across cohorts, which may reflect differences in sample composition, survey administration, response styles, healthcare contexts, or the meaning of the fair/poor categories across populations. Social engagement items also differed across cohorts. In addition, depressive symptoms were assessed using different versions of the CES‐D with varying cutoffs across cohorts, which may partly explain the observed heterogeneity in effect sizes. Therefore, the observed differences across cohorts should be interpreted with caution and not be attributed solely to cultural differences in susceptibility. Third, despite the longitudinal design supporting our proposed pathways, the possibility of reverse causality cannot be ruled out. Fourth, mediation effects were not fully consistent across sensitivity analyses. In complete case analysis, social engagement in CHARLS and cognitive function in HRS were no longer significant. These pathways should be interpreted with caution. Finally, the small number of included studies precluded formal assessment of publication bias, and meta‐regression could not be reliably performed.

In conclusion, findings from three prospective cohort studies and a meta‐analysis demonstrate that DSI is consistently associated with depressive symptoms in middle‐aged and older adults. In the primary analyses, both reduced social engagement and cognitive decline significantly mediated this association across all three cohorts, with comparable effect sizes. These mediation effects were largely consistent, although the mediating roles of social engagement in CHARLS and cognitive function in HRS were not robust in the complete case sensitivity analysis. Given that the measurements of social engagement were not identical across cohorts, these findings should be interpreted with caution, as they may partly reflect measurement heterogeneity rather than purely cultural differences. These findings suggest that routine screening for DSI, combined with interventions designed to maintain both social engagement and cognitive function, could help alleviate the public health burden of late‐life depressive symptoms. Future research using harmonized instruments across cultures is warranted to further validate these findings.

## Funding

This work was supported by the Ministry of Science and Technology Project of the People’s Republic of China (Grant 2023YFC3604605), the National Key Research and Development Program of China (Grant 2023YFC3605900), the Natural Science Foundation of Sichuan Province (Grant 2025ZNSFSC0018), and the National Clinical Research Center for Geriatrics, West China Hospital (Grant Z2023LC004).

## Disclosure

The funders were not involved in any aspect of the study, including its design, conduct, analysis, interpretation, or preparation of the manuscript.

## Conflicts of Interest

The authors declare no conflicts of interest.

## Supporting Information

Additional supporting information can be found online in the Supporting Information section.

## Supporting information


**Supporting Information** Figure S1: Data collection time points for the three studies. Figure S2: The enrollment flow diagrams in China Health and Retirement Longitudinal Study (CHARLS). Figure S3: The enrollment flow diagrams in Health and Retirement Study (HRS). Figure S4: The enrollment flow diagrams in English Longitudinal Study of Ageing (ELSA). Figure S5: The PRISMA flow diagram. Figure S6: The sensitivity analysis. (a) OR subgroup; (b) HR subgroup. Table S1: Social engagement assessed across three studies. Table S2: Chronic diseases assessed across three studies. Table S3: Six basic activities of daily living assessed across three studies. Table S4: Search strategies. Table S5: Correlation matrix of study variables. Table S6: Baseline characteristics of the participants in sensitivity analysis 1. Table S7: Association between DSI and depressive symptoms in sensitivity analysis 1. Table S8: Correlation analysis in sensitivity analysis 1. Table S9: Mediation roles of social engagement and cognitive function in sensitivity analysis 1. Table S10: Bootstrap analysis of the mediating model in sensitivity analysis 1. Table S11: Baseline characteristics of the participants in sensitivity analysis 2. Table S12: Association between DSI and depressive symptoms in sensitivity analysis 2. Table S13: Correlation analysis in sensitivity analysis 2. Table S14: Mediation roles of social engagement and cognitive function in sensitivity analysis 2. Table S15: Bootstrap analysis of the mediating model in sensitivity analysis 2. Table S16: Baseline characteristics of the participants in sensitivity analysis 3. Table S17: Association between DSI and depressive symptoms in sensitivity analysis 3. Table S18: Correlation analysis in sensitivity analysis 3. Table S19: Mediation roles of social engagement and cognitive function in sensitivity analysis 3. Table S20: Bootstrap analysis of the mediating model in sensitivity analysis 3. Table S21: Baseline characteristics of the participants in sensitivity analysis 4 for CHARLS. Table S22: Association between DSI and depressive symptoms in sensitivity analysis 4 for CHARLS. Table S23: Correlation analysis in sensitivity analysis 4 for CHARLS. Table S24: Mediation roles of social engagement and cognitive function in sensitivity analysis 4 for CHARLS. Table S25: Bootstrap analysis of the mediating model in sensitivity analysis 4 for CHARLS. Table S26: Characteristics of the included studies. Table S27: The Newcastle–Ottawa Scale assessment. Table S28: The subgroup analysis.

## Data Availability

The data for this analysis were obtained from publicly available datasets: CHARLS (http://charls.pku.edu.cn/), HRS (https://hrs.isr.umich.edu/), and ELSA (https://www.elsa-project.ac.uk/).
