# Supplementary material for: Dual Sensory Impairment and Depressive Symptoms: Association and Pathways in Three Cohorts and a Meta‐Analysis
Source: Depress Anxiety. 2026 Sep 29;2026:8488263. doi: 10.1155/da/8488263 (PMC13624546; doi:10.1155/da/8488263)
Supplement: Supplementary file 1 [file DA-2026-8488263-s001.docx]

**Supplementary materials**

[Figure S1. Data collection time points for the three studies	1](#_Toc213364338)

[Figure S2. The enrollment flow diagrams in China Health and Retirement Longitudinal Study (CHARLS)	2](#_Toc213364339)

[Figure S3. The enrollment flow diagrams in Health and Retirement Study (HRS)	2](#_Toc213364340)

[Figure S4. The enrollment flow diagrams in English Longitudinal Study of Ageing (ELSA)	2](#_Toc213364340)

[Table S1. Social engagement assessed across three studies	3](#_Toc213364341)

[Table S2. Chronic diseases assessed across three studies	5](#_Toc213364342)

[Table S3. Six basic activities of daily living assessed across three studies	6](#_Toc213364343)

[Table S4. Search strategies	7](#_Toc213364344)

[Table S5. Correlation matrix of study variables	8](#_Toc213364345)

[Table S6. Baseline characteristics of the participants in sensitivity analysis 1	9](#_Toc213364346)

[Table S7. Association between DSI and depressive symptoms in sensitivity analysis 1	10](#_Toc213364347)

[Table S8. Correlation analysis in sensitivity analysis 1	11](#_Toc213364348)

[Table S9. Mediation roles of social engagement and cognitive function in sensitivity analysis 1	12](#_Toc213364349)

[Table S10. Bootstrap analysis of the mediating model in sensitivity analysis 1	13](#_Toc213364350)

[Table S11. Baseline characteristics of the participants in sensitivity analysis 2	14](#_Toc213364351)

[Table S12. Association between DSI and depressive symptoms in sensitivity analysis 2	15](#_Toc213364352)

[Table S13. Correlation analysis in sensitivity analysis 2	16](#_Toc213364353)

[Table S14. Mediation roles of social engagement and cognitive function in sensitivity analysis 2	17](#_Toc213364354)

[Table S15. Bootstrap analysis of the mediating model in sensitivity analysis 2	18](#_Toc213364355)

[Table S16. Baseline characteristics of the participants in sensitivity analysis 3	19](#_Toc213364356)

[Table S17. Association between DSI and depressive symptoms in sensitivity analysis 3	20](#_Toc213364357)

[Table S18. Correlation analysis in sensitivity analysis 3	21](#_Toc213364358)

[Table S19. Mediation roles of social engagement and cognitive function in sensitivity analysis 3	22](#_Toc213364359)

[Table S20. Bootstrap analysis of the mediating model in sensitivity analysis 3	23](#_Toc213364360)

Table S21. Baseline characteristics of the participants in sensitivity analysis 4 for CHARLS..24

Table S22. Association between DSI and depressive symptoms in sensitivity analysis 4 for CHARLS……………………………………………………………………………………………….25

Table S23. Correlation analysis in sensitivity analysis 4 for CHARLS………………………….26

Table S24. Mediation roles of social engagement and cognitive function in sensitivity analysis 4 for CHARLS…………………………………………………………………………………………27

Table S25. Bootstrap analysis of the mediating model in sensitivity analysis 4 for CHARLS..28

Figure S5. The PRISMA flow diagram	……………………………………………………………..29

Table S26. Characteristics of the included studies………………………………………………..30

Table S27. The Newcastle-Ottawa Scale assessment…………………………………………...33

Figure S6. The sensitivity analysis………………………………………………………………….34

Table S28. The subgroup analysis………………………………………………………………….35

**Figure S1.** **Data collection time points for the three studies**

**Figure S2. The enrollment flow diagrams in China Health and Retirement Longitudinal Study (CHARLS)**

**Figure S3. The enrollment flow diagrams in Health and Retirement Study (HRS)**

**Figure S4.** **The enrollment flow diagrams in** **English Longitudinal Study of Ageing (ELSA)**

**Table S1. Social engagement assessed across three studies**

| **Population** | **Item** |
| --- | --- |
| China Health and Retirement Longitudinal Study | (1) Interacted with friends |
|  | (2) Played Ma-jong, played chess, played cards, or went to community club |
|  | (3) Provided help to family, friends, or neighbors who do not live with you and who did not pay you for the help |
|  | (4) Went to a sport, social, or other kind of club |
|  | (5) Took part in a community-related organization |
|  | (6) Done voluntary or charity work |
|  | (7) Cared for a sick or disabled adult who does not live with you and who did not pay you for the help |
|  | (8) Attended an educational or training course |
|  | (9) Stock investment |
|  | (10) Used the Internet |
|  | (11) Other |
|  | (12) None of these |
| Health and Retirement Study | (1) Care for a sick or disabled adult |
|  | (2) Do activities with grandchildren, nieces/nephews, or neighborhood children |
|  | (3) Do volunteer work with children or young people |
|  | (4) Do any other volunteer or charity work |
|  | (5) Attend an educational or training course |
|  | (6) Go to a sport, social, or other club |
|  | (7) Attend meetings of non-religious organizations, such as political, community, or other interest groups |
|  | (8) Pray privately in places other than a church or synagogue |
|  | (9) Read books, magazines, or newspapers |
|  | (10) Watch television |
|  | (11) Do word games such as crossword puzzles or Scrabble |
|  | (12) Play cards or games such as chess |
|  | (13) Do writing (such as letters, stories, or journal entries) |
|  | (14) Use a computer for e-mail, Internet or other tasks |
|  | (15) Do home or car maintenance or gardening |
|  | (16) Bake or cook something special |
|  | (17) Make clothes, knit, embroider, etc. |
|  | (18) Work on a hobby or project |
|  | (19) Play sports or exercise |
|  | (20) Walk for 20 minutes or more |
|  | (21) Participate in a local community arts group such as a choir, dance, photography, theatre, or music group |
| English Longitudinal Study of Ageing | (1) Go to the theatre, a concert or the opera |
|  | (2) Go to the cinema |
|  | (3) Go to art galleries or museums (4) Political party, trade union or environmental groups (5) Tenants groups, resident groups, neighborhood watch |
| **Population** | **Item** |
| English Longitudinal Study of Ageing | (6) Church or other religious groups |
|  | (7) Charitable associations |
|  | (8) Education, arts or music groups or evening classes |
|  | (9) Social Clubs |
|  | (10) Sports clubs, gyms, exercise classes |
|  | (11) Any other organizations, clubs or societies |

**Table S2. Chronic diseases assessed across three studies**

| **Population** | **Item** |
| --- | --- |
| China Health and Retirement Longitudinal Study | (1) Hypertension |
|  | (2) Dyslipidemia (elevation of low density lipoprotein, triglycerides (TGs), and total cholesterol, or a low high density lipoprotein level) |
|  | (3) Diabetes or high blood sugar |
|  | (4) Cancer or malignant tumor (excluding minor skin cancers) |
|  | (5) Chronic lung diseases, such as chronic bronchitis, emphysema (excluding tumors, or cancer) |
|  | (6) Liver disease (except fatty liver, tumors, and cancer) |
|  | (7) Heart attack, coronary heart disease, angina, congestive heart failure, or other heart problems |
|  | (8) Stroke |
|  | (9) Kidney disease (except for tumor or cancer) |
|  | (10) Stomach or other digestive disease (except for tumor or cancer) |
|  | (11) Emotional, nervous, or psychiatric problems |
|  | (12) Memory-related disease (e.g., Alzheimer's disease) |
|  | (13) Arthritis or rheumatism |
|  | (14) Asthma |
| Health and Retirement Study | (1) High blood pressure or hypertension |
|  | (2) Diabetes or high blood sugar |
|  | (3) Cancer or a malignant tumor of any kind except skin cancer |
|  | (4) Chronic lung disease except asthma such as chronic bronchitis or emphysema |
|  | (5) Heart attack, coronary heart disease, angina, congestive heart failure, or other heart problems |
|  | (6) Stroke or transient ischemic attack (TIA) |
|  | (7) Emotional, nervous, or psychiatric problems |
|  | (8) Arthritis or rheumatism |
|  | (9) Memory-related disease (Alzheimer's disease or dementia) |
| English Longitudinal Study of Ageing | (1) Blood pressure or hypertension |
|  | (2) High cholesterol |
|  | (3) Diabetes or high blood sugar |
|  | (4) Cancer or a malignant tumor (excluding minor skin cancers) |
|  | (5) Chronic lung disease such as chronic bronchitis or emphysema |
|  | (6) Heart problems: angina, a heart attack (including myocardial infarction or coronary thrombosis), congestive heart failure, a heart murmur, an abnormal heart rhythm, or any other heart trouble |
|  | (7) Stroke (cerebrovascular disease) |
|  | (8) Emotional, nervous, or psychiatric problems |
|  | (9) Arthritis (including osteoarthritis or rheumatism). |
|  | (10) Asthma |
|  | (11) Cataracts |
|  | (12) Parkinson's disease (13) Memory-related disease (Alzheimer's disease or dementia) |

**Table S3. Six basic activities of daily living** **assessed across three studies**

| **Population** | **Item** |
| --- | --- |
| CHARLS | dressing, bathing and showering, eating, getting in and out of bed, using the toilet, and controlling urination and defecation |
| HRS | walking across a room, dressing, bathing, eating, getting in and out of bed, and using the toilet |
| ELSA | walking across a room, dressing, bathing, eating, getting in and out of bed, and using the toilet |

*Notes*: *CHARLS* China Health and Retirement Longitudinal Study, *HRS* Health and Retirement Study, *ELSA* English Longitudinal Study of Ageing

**Table S4.** **Search strategies**

| **Database** | **Search strategies** | **Results** |
| --- | --- | --- |
| Pubmed | #1 dual sensory impairment[Title/Abstract] OR sensory impairment[Title/Abstract] OR "Hearing Loss"[Mesh] OR "Vision Disorders"[Mesh] OR hearing impairment[Title/Abstract] OR vision impairment[Title/Abstract] OR vision loss[Title/Abstract] OR visual impairment[Title/Abstract] OR visual loss[Title/Abstract] | 1281 |
|  | #2 "Depressive Disorder"[Mesh] OR "Depression"[Mesh] OR depressive symptoms[Title/Abstract] |  |
|  | #3 #1 AND #2 |  |
| Web of Science | #1 (((((((AB=("dual sensory impairment")) OR AB=("sensory impairment")) OR AB=("hearing loss")) OR AB=("hearing impairment")) OR AB=("vision loss")) OR AB=("vision impairment")) OR AB=("visual loss")) OR AB=("visual impairment") | 2022 |
|  | #2 AB=("depressive symptoms") OR AB=("depression") |  |
|  | #3 #1 AND #2 |  |
| Embase and Medline via Ovid | #1 "dual sensory impairment".mp. | Embase: 628  Medline: 244 |
|  | #2 *hearing impairment/ |  |
|  | #3 *visual impairment/ |  |
|  | #4 "depressive symptoms".mp. |  |
|  | #5 *depression/ |  |
|  | #6 #1 OR #2 OR #3 |  |
|  | #7 #4 OR #5 |  |
|  | #8 #6 AND #7 |  |

**Table S5.** **Correlation matrix of study variables**

| **Population** | **Variable** | **DSI** | **Social engagement** | **Cognitive function** | **Depressive symptoms** |
| --- | --- | --- | --- | --- | --- |
| CHARLS | DSI | 1 |  |  |  |
|  | SE | -0.0704^***^ | 1 |  |  |
|  | Cognitive function | -0.0747^***^ | 0.2718^***^ | 1 |  |
|  | Depressive symptoms | 0.0672^***^ | -0.1048^***^ | -0.1561^***^ | 1 |
| HRS | DSI | 1 |  |  |  |
|  | SE | -0.0903^***^ | 1 |  |  |
|  | Cognitive function | -0.1461^***^ | 0.2382^***^ | 1 |  |
|  | Depressive symptoms | 0.0817^***^ | -0.0720^***^ | -0.1224^***^ | 1 |
| ELSA | DSI | 1 |  |  |  |
|  | SE | -0.0614^***^ | 1 |  |  |
|  | Cognitive function | -0.0971^***^ | 0.2661^***^ | 1 |  |
|  | Depressive symptoms | 0.1125^***^ | -0.0847^***^ | -0.0884^***^ | 1 |

*Notes*: CHARLS = China Health and Retirement Longitudinal Study; HRS = Health and Retirement Study; ELSA = English Longitudinal Study of Ageing; DSI = dual sensory impairment.

^***^*P* < 0.001

**Sensitivity analysis 1, exclude participants with missing covariates**

**Table S6. Baseline characteristics of the participants in sensitivity analysis 1**

| **Characteristics ^a^** | **CHARLS**  **(n = 3861)** | **HRS**  **(n = 1940)** | **ELSA**  **(n = 3625)** |
| --- | --- | --- | --- |
| Age (year) | 60.0 ± 6.7 | 71.0 ± 6.7 | 64.9 ± 7.9 |
| Women | 1698 (44.0) | 1110 (57.2) | 1917(52.9) |
| Rural resident | 2250 (58.3) | / | / |
| Caucasian | / | 1606 (82.8) | 3542 (97.7) |
| High level of education | 573 (14.8) | 1643 (84.7) | 2975 (82.1) |
| Married | 3395 (87.9) | 1254 (64.6) | 2689 (74.2) |
| Smoking | 1349 (34.9) | 165 (8.5) | 294 (8.1) |
| Drinking | 1330 (34.5) | 797 (41.1) | 2645 (73.0) |
| No. of chronic diseases |  |  |  |
| 0 | 1381 (35.8) | 185 (9.5) | 744 (20.5) |
| 1 | 1198 (31.0) | 501 (25.8) | 1036 (28.6) |
| ≥ 2 | 1282 (33.2) | 1254 (64.7) | 1845 (50.9) |
| ADL disability | 316 (8.2) | 166 (8.6) | 325 (9.0) |
| DSI | 1689 (43.8) | 103 (5.3) | 78 (2.2) |
| Depressive symptoms | 6 (3, 10) | 0 (0, 1) | 0 (0, 1) |

*Notes*: *CHARLS* China Health and Retirement Longitudinal Study, *HRS* Health and Retirement Study, *ELSA* English Longitudinal Study of Ageing, *SD* standard deviation, *IQR* interquartile range, *ADL* activity of daily living, *DSI* dual sensory impairment

^a^ Continuous variables are presented as mean ± standard deviation (SD) or median (interquartile range). Categorical variables are presented as number (percentage).

**Table S7. Association between DSI and depressive symptoms** **in sensitivity analysis 1**

| **Model** | **Population** | **OR** | **Lower 95% CI** | **Upper 95% CI** | ***P*** |
| --- | --- | --- | --- | --- | --- |
| Model 1 | CHARLS | 1.2205 | 1.052739 | 1.414995 | 0.008 |
|  | HRS | 3.501289 | 2.211597 | 5.543064 | < 0.001 |
|  | ELSA | 2.257188 | 1.286751 | 3.959507 | 0.005 |
| Model 2 | CHARLS | 1.221077 | 1.053195 | 1.41572 | 0.008 |
|  | HRS | 3.331592 | 2.097369 | 5.292111 | < 0.001 |
|  | ELSA | 2.277836 | 1.295989 | 4.003534 | 0.0054 |
| Model 3 | CHARLS | 1.164888 | 1.002942 | 1.352983 | 0.046 |
|  | HRS | 3.134503 | 1.960405 | 5.011776 | < 0.001 |
|  | ELSA | 1.91389 | 1.07946 | 3.39334 | 0.026 |

*Notes*: *CHARLS* China Health and Retirement Longitudinal Study, *HRS* Health and Retirement Study, *ELSA* English Longitudinal Study of Ageing, *OR* odds ratio, *CI* confidence interval

**Table S8. Correlation analysis** **in sensitivity analysis 1**

| **Population** | **Variable** | **DSI** | **Social engagement** | **Cognitive function** | **Depressive symptoms** |
| --- | --- | --- | --- | --- | --- |
| CHARLS | DSI | 1 |  |  |  |
|  | SE | -0.0491^**^ | 1 |  |  |
|  | Cognitive function | -0.0894^***^ | 0.2080^***^ | 1 |  |
|  | Depressive symptoms | 0.0586^***^ | -0.1033^***^ | -0.1591^***^ | 1 |
| HRS | DSI | 1 |  |  |  |
|  | SE | -0.1614^***^ | 1 |  |  |
|  | Cognitive function | -0.1859^***^ | 0.3585^***^ | 1 |  |
|  | Depressive symptoms | 0.1567^***^ | -0.1607^***^ | -0.1132^***^ | 1 |
| ELSA | DSI | 1 |  |  |  |
|  | SE | -0.0687^***^ | 1 |  |  |
|  | Cognitive function | -0.0843^***^ | 0.2841^***^ | 1 |  |
|  | Depressive symptoms | 0.0648^***^ | -0.0927^***^ | -0.1198^***^ | 1 |

*Notes*: *CHARLS* China Health and Retirement Longitudinal Study, *HRS* Health and Retirement Study, *ELSA* English Longitudinal Study of Ageing, *DSI* dual sensory impairment

^***^*P* < 0.001

^**^*P* < 0.01

**Table S9. Mediation roles of social engagement and cognitive function in sensitivity analysis 1**

| **Population** | **Dependent variable** | **Independent variable** | ***β*** | **Lower 95% CI** | **Upper 95% CI** | ***t*** | ***P*** |
| --- | --- | --- | --- | --- | --- | --- | --- |
| CHARLS | M1 | X | -0.121 | -0.273 | 0.031 | -1.5601 | 0.1188 |
|  | M2 | X | -0.432 | -0.7 | -0.1639 | -3.1602 | 0.0016 |
|  | Y | X | 0.2791 | -0.068 | 0.6263 | 1.5758 | 0.1152 |
|  | Y | M1 | -0.141 | -0.213 | -0.0677 | -3.7828 | 0.0002 |
|  | Y | M2 | -0.132 | -0.173 | -0.0905 | -6.2558 | < 0.001 |
| HRS | M1 | X | -6.2873 | -9.0577 | -3.5168 | -4.4508 | < 0.001 |
|  | M2 | X | -1.6956 | -2.3922 | -0.999 | -4.7734 | < 0.001 |
|  | Y | X | 0.8123 | 0.5162 | 1.1084 | 5.3802 | < 0.001 |
|  | Y | M1 | -0.0112 | -0.0161 | -0.0064 | -4.5419 | < 0.001 |
|  | Y | M2 | -0.0137 | -0.0329 | 0.0056 | -1.3906 | 0.1645 |
| ELSA | M1 | X | -1.2251 | -2.0559 | -0.3943 | -2.8911 | 0.0039 |
|  | M2 | X | -1.2328 | -1.9902 | -0.4754 | -3.1913 | 0.0014 |
|  | Y | X | 0.4079 | 0.1104 | 0.7054 | 2.6882 | 0.0072 |
|  | Y | M1 | -0.0136 | -0.0255 | -0.0018 | -2.2534 | 0.0243 |
|  | Y | M2 | -0.0279 | -0.0409 | -0.0149 | -4.2032 | < 0.001 |

*Notes*: *CHARLS* China Health and Retirement Longitudinal Study, *HRS* Health and Retirement Study, *ELSA* English Longitudinal Study of Ageing, *CI* confidence interval

*X* dual sensory impairment, *M1* social engagement, *M2* cognitive function, *Y* depressive symptoms

**Table S10. Bootstrap analysis of the mediating model in sensitivity analysis 1**

| **Population** | **Path** | **Effect** | **SE** | **95% CI** | | **Mediated proportion (%)** |
| --- | --- | --- | --- | --- | --- | --- |
|  |  |  |  | **Lower** | **Upper** |  |
| CHARLS | Total effect | 0.353 | 0.1782 | 0.0036 | 0.7023 | - |
|  | Direct effect | 0.2791 | 0.1771 | -0.0681 | 0.6263 | 79 |
|  | Total indirect effect | 0.0739 | 0.0239 | 0.0297 | 0.1245 | 21 |
|  | Ind1, X→M1→Y | 0.017 | 0.0116 | -0.0028 | 0.0426 | - |
|  | Ind2, X→M2→Y | 0.0569 | 0.02 | 0.0196 | 0.0989 | 16 |
|  | Ind1 - ind2 | -0.0399 | 0.0223 | -0.0841 | 0.0016 | - |
| HRS | Total effect | 0.906 | 0.1506 | 0.6107 | 1.2013 | - |
|  | Direct effect | 0.8123 | 0.151 | 0.5162 | 1.1084 | 90 |
|  | Total indirect effect | 0.0937 | 0.029 | 0.0409 | 0.1538 | 10 |
|  | Ind1, X→M1→Y | 0.0705 | 0.0239 | 0.0281 | 0.1217 | 8 |
|  | Ind2, X→M2→Y | 0.0232 | 0.02 | -0.0134 | 0.0668 | - |
|  | Ind1 - ind2 | 0.0474 | 0.0331 | -0.0154 | 0.1169 | - |
| ELSA | Total effect | 0.4589 | 0.1519 | 0.161 | 0.7568 | - |
|  | Direct effect | 0.4079 | 0.1517 | 0.1104 | 0.7054 | 89 |
|  | Total indirect effect | 0.051 | 0.0182 | 0.0196 | 0.091 | 11 |
|  | Ind1, X→M1→Y | 0.0167 | 0.0093 | 0.0013 | 0.0377 | 4 |
|  | Ind2, X→M2→Y | 0.0343 | 0.0155 | 0.0091 | 0.0686 | 7 |
|  | Ind1 - ind2 | -0.0177 | 0.0179 | -0.0559 | 0.0147 | - |

*Notes*: *CHARLS* China Health and Retirement Longitudinal Study, *HRS* Health and Retirement Study, *ELSA* English Longitudinal Study of Ageing, *SE* standard error, *CI* confidence interval

*X* dual sensory impairment, *M1* social engagement, *M2* cognitive function, *Y* depressive symptoms

**Sensitivity analysis 2, exclude participants with baseline ADL disability**

**Table S11. Baseline characteristics of the participants in sensitivity analysis 2**

| **Characteristics ^a^** | **CHARLS**  **(n = 4630)** | **HRS**  **(n =8375)** | **ELSA**  **(n = 4568)** |
| --- | --- | --- | --- |
| Age (year) | 60. 0 ± 6.8 | 65.4 ± 9.2 | 65.0 ± 8.1 |
| Women | 2088 (45.1) | 4816 (57.7) | 2458(53.8) |
| Rural resident | 2779 (60.0) | / | / |
| Caucasian | / | 6251 (74.6) | 4440 (97.2) |
| High level of education | 629 (13.6) | 6979(83.3) | 3537 (77.4) |
| Married | 4032 (87.1) | 5483 (65.5) | 3315 (72.6) |
| Smoking | 1616 (34.9) | 922 (11.0) | 420 (9.2) |
| Drinking | 1579 (34.1) | 3680 (43.9) | 3232 (70.8) |
| No. of chronic diseases |  |  |  |
| 0 | 1729 (37.3) | 1613 (19.3) | 981 (21.5) |
| 1 | 1477 (31.9) | 2359 (28.2) | 1315 (28.8) |
| ≥ 2 | 1424 (30.8) | 4403 (52.5) | 2272 (49.7) |
| DSI | 1994 (43.1) | 447 (5.3) | 86 (1.9) |
| Depressive symptoms | 4 (2, 7) | 0 (0, 1) | 0 (0, 1) |

*Notes*: *CHARLS* China Health and Retirement Longitudinal Study, *HRS* Health and Retirement Study, *ELSA* English Longitudinal Study of Ageing, *DSI* dual sensory impairment

^a^ Continuous variables are presented as mean ± standard deviation (SD) or median (interquartile range). Categorical variables are presented as number (percentage).

**Table S12. Association between DSI and depressive symptoms in sensitivity analysis 2**

| **Model** | **Population** | **OR** | **Lower 95% CI** | **Upper 95% CI** | ***P*** |
| --- | --- | --- | --- | --- | --- |
| Model 1 | CHARLS | 1.286744 | 1.125182 | 1.471504 | < 0.001 |
|  | HRS | 1.918297 | 1.490175 | 2.469415 | < 0.001 |
|  | ELSA | 2.697127 | 1.623434 | 4.480931 | < 0.001 |
| Model 2 | CHARLS | 1.286293 | 1.124745 | 1.471043 | < 0.001 |
|  | HRS | 1.904551 | 1.479063 | 2.452441 | < 0.001 |
|  | ELSA | 2.697306 | 1.618943 | 4.493957 | < 0.001 |
| Model 3 | CHARLS | 1.249899 | 1.092 | 1.43063 | 0.001 |
|  | HRS | 1.841678 | 1.427956 | 2.375268 | < 0.001 |
|  | ELSA | 2.549751 | 1.528903 | 4.252217 | < 0.001 |

*Notes*: *CHARLS* China Health and Retirement Longitudinal Study, *HRS* Health and Retirement Study, *ELSA* English Longitudinal Study of Ageing, *OR* odds ratio, *CI* confidence interval

**Table S13. Correlation analysis in sensitivity analysis 2**

| **Population** | **Variable** | **DSI** | **Social engagement** | **Cognitive function** | **Depressive symptoms** |
| --- | --- | --- | --- | --- | --- |
| CHARLS | DSI | 1 |  |  |  |
|  | SE | -0.0704^***^ | 1 |  |  |
|  | Cognitive function | -0.0747^***^ | 0.2718^***^ | 1 |  |
|  | Depressive symptoms | 0.0672^***^ | -0.1048^***^ | -0.1561^***^ | 1 |
| HRS | DSI | 1 |  |  |  |
|  | SE | -0903^***^ | 1 |  |  |
|  | Cognitive function | -0.1461^***^ | 0.2382*** | 1 |  |
|  | Depressive symptoms | 0.0817^***^ | -0.0720*** | -0.1224*** | 1 |
| ELSA | DSI | 1 |  |  |  |
|  | SE | -0.0641^***^ | 1 |  |  |
|  | Cognitive function | -0.0971^***^ | 0.2661^***^ | 1 |  |
|  | Depressive symptoms | 0.1125^***^ | -0.0847^***^ | -0.0884^***^ | 1 |

*Notes*: *CHARLS* China Health and Retirement Longitudinal Study, *HRS* Health and Retirement Study, *ELSA* English Longitudinal Study of Ageing, *DSI* dual sensory impairment

^***^*P* < 0.001

**Table S14. Mediation roles of social engagement and cognitive function in sensitivity analysis 2**

| **Population** | **Dependent variable** | **Independent variable** | ***β*** | **Lower 95% CI** | **Upper 95% CI** | ***t*** | ***P*** |
| --- | --- | --- | --- | --- | --- | --- | --- |
| CHARLS | M1 | X | -0.1553 | -0.29 | -0.0203 | -2.2553 | 0.0242 |
|  | M2 | X | -0.4989 | -0.753 | -0.2447 | -3.8482 | 0.0001 |
|  | Y | X | 0.4526 | 0.1339 | 0.7712 | 2.7846 | 0.0054 |
|  | Y | M1 | -0.1219 | -0.191 | -0.053 | -3.4706 | 0.0005 |
|  | Y | M2 | -0.1366 | -0.173 | -0.1 | -7.3198 | < 0.001 |
| HRS | M1 | X | -6.3282 | -7.6276 | -5.0288 | -9.5464 | < 0.001 |
|  | M2 | X | -1.3607 | -1.7203 | -1.0011 | -7.4177 | < 0.001 |
|  | Y | X | 0.3528 | 0.2143 | 0.4913 | 4.9925 | < 0.001 |
|  | Y | M1 | -0.005 | -0.0073 | -0.0027 | -4.2255 | < 0.001 |
|  | Y | M2 | -0.0185 | -0.0268 | -0.0102 | -4.3544 | < 0.001 |
| ELSA | M1 | X | -0.971 | -1.7621 | -0.1798 | -2.4061 | 0.0162 |
|  | M2 | X | -1.3489 | -2.1261 | -0.5717 | -3.4025 | 0.0007 |
|  | Y | X | 0.6414 | 0.3555 | 0.9272 | 4.399 | < 0.001 |
|  | Y | M1 | -0.0151 | -0.0258 | -0.0045 | -2.7838 | 0.0054 |
|  | Y | M2 | -0.0246 | -0.0354 | -0.0137 | -4.441 | < 0.001 |

*Notes*: *CHARLS* China Health and Retirement Longitudinal Study, *HRS* Health and Retirement Study, *ELSA* English Longitudinal Study of Ageing, *CI* confidence interval

*X* dual sensory impairment, *M1* social engagement, *M2* cognitive function, *Y* depressive symptoms

**Table S15. Bootstrap analysis of the mediating model in sensitivity analysis 2**

| **Population** | **Path** | **Effect** | **SE** | **95% CI** | | **Mediated proportion (%)** |
| --- | --- | --- | --- | --- | --- | --- |
|  |  |  |  | **Lower** | **Upper** |  |
| CHARLS | Total effect | 0.5396 | 0.1635 | 0.2191 | 0.8602 | - |
|  | Direct effect | 0.4526 | 0.1625 | 0.1339 | 0.7712 | 84 |
|  | Total indirect effects | 0.0871 | 0.0229 | 0.0444 | 0.1334 | 16 |
|  | Ind1, X→M1→Y | 0.0189 | 0.0098 | 0.0026 | 0.0407 | 4 |
|  | Ind2, X→M2→Y | 0.0681 | 0.0201 | 0.0316 | 0.1104 | 13 |
|  | Ind1 - ind2 | -0.0492 | 0.0219 | -0.0941 | -0.0085 | - |
| HRS | Total effect | 0.4094 | 0.0703 | 0.2716 | 0.5473 | - |
|  | Direct effect | 0.3528 | 0.0707 | 0.2143 | 0.4913 | 86 |
|  | Total indirect effects | 0.0566 | 0.0105 | 0.0374 | 0.0786 | 14 |
|  | Ind1, X→M1→Y | 0.0314 | 0.0083 | 0.0159 | 0.0485 | 8 |
|  | Ind2, X→M2→Y | 0.0252 | 0.0071 | 0.0122 | 0.0403 | 6 |
|  | Ind1 - ind2 | 0.0063 | 0.0114 | -0.0163 | 0.0286 | - |
| ELSA | Total effect | 0.6892 | 0.1461 | 0.4029 | 0.9756 | - |
|  | Direct effect | 0.6414 | 0.1458 | 0.3555 | 0.9272 | 93 |
|  | Total indirect effects | 0.0479 | 0.0149 | 0.0212 | 0.079 | 7 |
|  | Ind1, X→M1→Y | 0.0147 | 0.0078 | 0.0023 | 0.0329 | 2 |
|  | Ind2, X→M2→Y | 0.0332 | 0.0128 | 0.011 | 0.0606 | 5 |
|  | Ind1 - ind2 | -0.0185 | 0.0151 | -0.05 | 0.0106 | - |

*Notes*: *CHARLS* China Health and Retirement Longitudinal Study, *HRS* Health and Retirement Study, *ELSA* English Longitudinal Study of Ageing, *SE* standard error, *CI* confidence interval

*X* dual sensory impairment, *M1* social engagement, *M2* cognitive function, *Y* depressive symptoms

**Sensitivity analysis 3, exclude individuals with baseline emotional, nervous, psychiatric, or memory-related diseases**

**Table S16. Baseline characteristics of the participants in sensitivity analysis 3**

| **Characteristics ^a^** | **CHARLS**  **(n = 5010)** | **HRS**  **(n = 8095)** | **ELSA**  **(n = 4688)** |
| --- | --- | --- | --- |
| Age (year) | 60.2 ± 6.9 | 65.7 ± 9.4 | 65.5 ± 8.3 |
| Women | 2295 (45.8) | 4534 (56.0) | 2496 (53.2) |
| Rural resident | 3037 (60.6) | / | / |
| Caucasian | / | 5903 (72.9) | 4556 (97.2) |
| High level of education | 659 (13.2) | 6661 (82.3) | 3544 (75.6) |
| Married | 4351 (86.8) | 5291 (65.4) | 3409 (72.7) |
| Smoking | 1723 (34.4) | 874 (10.8) | 430 (9.2) |
| Drinking | 1673 (33.4) | 3522 (43.5) | 3275 (69.9) |
| No. of chronic diseases |  |  |  |
| 0 | 1810 (36.1) | 1646 (20.3) | 1008 (21.5) |
| 1 | 1576 (31.4) | 2372(29.3) | 1329 (28.3) |
| ≥ 2 | 1624 (32.5) | 4077 (50.4) | 2351(50.2) |
| ADL disability | 431 (8.6) | 245 (7.9) | 326 (8.8) |
| DSI | 2226 (44.4) | 449 (5.5) | 108 (2.3) |
| Depressive symptoms | 4 (2, 7) | 0 (0, 1) | 0 (0, 1) |

*Notes*: *CHARLS* China Health and Retirement Longitudinal Study, *HRS* Health and Retirement Study, *ELSA* English Longitudinal Study of Ageing, *ADL* Activity of Daily Living, *DSI* dual sensory impairment

^a^ Continuous variables are presented as mean ± standard deviation (SD) or median (interquartile range). Categorical variables are presented as number (percentage).

**Table S17. Association between DSI and depressive symptoms in sensitivity analysis 3**

| **Model** | **Population** | **OR** | **Lower 95% CI** | **Upper 95% CI** | ***P*** |
| --- | --- | --- | --- | --- | --- |
| Model 1 | CHARLS | 1.246362 | 1.096163 | 1.417141 | 0.001 |
|  | HRS | 2.109292 | 1.639263 | 2.714094 | < 0.001 |
|  | ELSA | 1.211907 | 1.064999 | 1.37908 | < 0.001 |
| Model 2 | CHARLS | 1.24651 | 1.096258 | 1.417356 | 0.001 |
|  | HRS | 2.088113 | 1.622441 | 2.687441 | < 0.001 |
|  | ELSA | 2.070548 | 1.607821 | 2.666446 | < 0.001 |
| Model 3 | CHARLS | 1.211907 | 1.064999 | 1.37908 | 0.004 |
|  | HRS | 2.070548 | 1.607821 | 2.666446 | < 0.001 |
|  | ELSA | 2.214368 | 1.400486 | 3.501233 | 0.001 |

*Notes*: *CHARLS* China Health and Retirement Longitudinal Study, *HRS* Health and Retirement Study, *ELSA* English Longitudinal Study of Ageing, *OR* odds ratio, *CI* confidence interval

**Table S18. Correlation analysis in sensitivity analysis 3**

| **Population** | **Variable** | **DSI** | **Social engagement** | **Cognitive function** | **Depressive symptoms** |
| --- | --- | --- | --- | --- | --- |
| CHARLS | DSI | 1 |  |  |  |
|  | SE | -0.0704^***^ | 1 |  |  |
|  | Cognitive function | -0.0747^***^ | 0.2718^***^ | 1 |  |
|  | Depressive symptoms | 0.0672^***^ | -0.1048^***^ | -0.1561^***^ | 1 |
| HRS | DSI | 1 |  |  |  |
|  | SE | -0.0903^***^ | 1 |  |  |
|  | Cognitive function | -0.1461^***^ | 0.2382^***^ | 1 |  |
|  | Depressive symptoms | 0.0817^***^ | -0.0720^***^ | -0.1224^***^ | 1 |
| ELSA | DSI | 1 |  |  |  |
|  | SE | -0.0641^***^ | 1 |  |  |
|  | Cognitive function | -0.0971^***^ | 0.2661^***^ | 1 |  |
|  | Depressive symptoms | 0.1125^***^ | -0.0847^***^ | -0.0884^***^ | 1 |

*Notes*: *CHARLS* China Health and Retirement Longitudinal Study, *HRS* Health and Retirement Study, *ELSA* English Longitudinal Study of Ageing, *DSI* dual sensory impairment

^***^*P* < 0.001

**Table S19. Mediation roles of social engagement and cognitive function in sensitivity analysis 3**

| **Population** | **Dependent variable** | **Independent variable** | ***β*** | **Lower 95% CI** | **Upper 95% CI** | ***t*** | ***P*** |
| --- | --- | --- | --- | --- | --- | --- | --- |
| CHARLS | M1 | X | -0.1695 | -0.2982 | -0.0407 | -2.5797 | 0.0099 |
|  | M2 | X | -0.4676 | -0.7131 | -0.2222 | -3.735 | 0.0002 |
|  | Y | X | 0.3818 | 0.0721 | 0.6914 | 2.4172 | 0.0157 |
|  | Y | M1 | -0.1402 | -0.2077 | -0.0727 | -4.0707 | < 0.001 |
|  | Y | M2 | -0.1346 | -0.17 | -0.0992 | -7.4504 | < 0.001 |
| HRS | M1 | X | -5.8939 | -7.196 | -4.5918 | -8.8732 | < 0.001 |
|  | M2 | X | -1.4234 | -1.7841 | -1.0627 | -7.7357 | < 0.001 |
|  | Y | X | 0.4299 | 0.2979 | 0.5619 | 6.3849 | < 0.001 |
|  | Y | M1 | -0.0037 | -0.006 | -0.0015 | -3.2894 | 0.001 |
|  | Y | M2 | -0.0216 | -0.0296 | -0.0135 | -5.246 | < 0.001 |
| ELSA | M1 | X | -1.12 | -1.8294 | -0.4106 | -3.0952 | 0.002 |
|  | M2 | X | -1.1236 | -1.8252 | -0.422 | -3.1398 | 0.0017 |
|  | Y | X | 0.4646 | 0.2079 | 0.7213 | 3.548 | 0.0004 |
|  | Y | M1 | -0.0128 | -0.0233 | -0.0023 | -2.388 | 0.017 |
|  | Y | M2 | -0.0282 | -0.0388 | -0.0176 | -5.1937 | < 0.001 |

*Notes*: *CHARLS* China Health and Retirement Longitudinal Study, *HRS* Health and Retirement Study, *ELSA* English Longitudinal Study of Ageing, *CI* confidence interval

*X* dual sensory impairment, *M1* social engagement, *M2* cognitive function, *Y* depressive symptoms

**Table S20. Bootstrap analysis of the mediating model in sensitivity analysis 3**

| **Population** | **Path** | **Effect** | **SE** | **95% CI** | | **Mediated proportion (%)** |
| --- | --- | --- | --- | --- | --- | --- |
|  |  |  |  | **Lower** | **Upper** |  |
| CHARLS | Total effect | 0.4685 | 0.159 | 0.157 | 0.7801 | - |
|  | Direct effect | 0.3818 | 0.1579 | 0.072 | 0.6914 | 81 |
|  | Total indirect effects | 0.0867 | 0.0224 | 0.044 | 0.1324 | 19 |
|  | Ind1, X→M1→Y | 0.0238 | 0.0106 | 0.005 | 0.0473 | 5 |
|  | Ind2, X→M2→Y | 0.0629 | 0.019 | 0.027 | 0.1018 | 13 |
|  | Ind1 - ind2 | -0.0392 | 0.0212 | -0.082 | 0.0008 | - |
| HRS | Total effect | 0.4826 | 0.067 | 0.3513 | 0.614 | - |
|  | Direct effect | 0.4299 | 0.0673 | 0.2979 | 0.5619 | 89 |
|  | Total indirect effects | 0.0528 | 0.01 | 0.0348 | 0.0737 | 11 |
|  | Ind1, X→M1→Y | 0.0221 | 0.0072 | 0.009 | 0.0369 | 5 |
|  | Ind2, X→M2→Y | 0.0307 | 0.0074 | 0.0175 | 0.046 | 6 |
|  | Ind1 - ind2 | -0.0086 | 0.0106 | -0.0295 | 0.0123 | - |
| ELSA | Total effect | 0.5107 | 0.1312 | 0.2534 | 0.7679 | - |
|  | Direct effect | 0.4646 | 0.131 | 0.2079 | 0.7213 | 91 |
|  | Total indirect effects | 0.046 | 0.015 | 0.0188 | 0.0779 | 9 |
|  | Ind1, X→M1→Y | 0.0144 | 0.0076 | 0.0019 | 0.0314 | 3 |
|  | Ind2, X→M2→Y | 0.0317 | 0.0127 | 0.0097 | 0.0583 | 6 |
|  | Ind1 - ind2 | -0.0173 | 0.0146 | -0.047 | 0.0099 | - |

*Notes*: *CHARLS* China Health and Retirement Longitudinal Study, *HRS* Health and Retirement Study, *ELSA* English Longitudinal Study of Ageing, *SE* standard error, *CI* confidence interval

*X* dual sensory impairment, *M1* social engagement, *M2* cognitive function, *Y* depressive symptoms

**Sensitivity analysis 4, treat education as a multi-categorical variable in CHARLS**

**Table S21. Baseline characteristics of the participants in sensitivity analysis 4 for CHARLS**

| **Characteristics ^a^** | **CHARLS**  **(n = 5076)** |
| --- | --- |
| Age (year) | 60.2 ± 6.9 |
| Women | 2320 (45.7) |
| Rural resident | 3077 (60.6) |
| Caucasian | / |
| Education level |  |
| Illiteracy | 1271 (25.0) |
| Primary school | 2127 (41.9) |
| Middle school | 1014 (20.0) |
| High level of education | 664 (13.1) |
| Married | 4406 (86.8) |
| Smoking | 1748 (34.4) |
| Drinking | 1692 (33.3) |
| No. of chronic diseases |  |
| 0 | 1811 (35.7) |
| 1 | 1595 (31.4) |
| ≥ 2 | 1671 (32.9) |
| ADL disability | 446 (8.8) |
| DSI | 2257 (44.5) |
| Depressive symptoms | 4 (2, 7) |

*Notes*: *CHARLS* China Health and Retirement Longitudinal Study, *ADL* Activity of Daily Living, *DSI* dual sensory impairment

^a^ Continuous variables are presented as mean ± standard deviation (SD) or median (interquartile range). Categorical variables are presented as number (percentage).

**Table S22. Association between DSI and depressive symptoms in sensitivity analysis 4 for CHARLS**

| **Model** | **OR** | **Lower 95% CI** | **Upper 95% CI** | | ***P*** | |
| --- | --- | --- | --- | --- | --- | --- |
| Model 1 | 1.25318 | 1.103626 | | 1.423001 | | 0.001 |
| Model 2 | 1.252688 | 1.103158 | | 1.422487 | | 0.001 |
| Model 3 | 1.20085 | 1.056069 | | 1.365479 | | 0.005 |

*Notes*: *CHARLS* China Health and Retirement Longitudinal Study, *OR* odds ratio, *CI* confidence interval

**Table S23. Correlation analysis in sensitivity analysis 4 for CHARLS**

| **Variable** | **DSI** | **Social engagement** | **Cognitive function** | **Depressive symptoms** |
| --- | --- | --- | --- | --- |
| DSI | 1 |  |  |  |
| SE | -0.0704^***^ | 1 |  |  |
| Cognitive function | -0.0747^***^ | 0.2718^***^ | 1 |  |
| Depressive symptoms | 0.0672^***^ | -0.1048^***^ | -0.1561^***^ | 1 |

*Notes*: *DSI* dual sensory impairment

^***^*P* < 0.001

**Table S24. Mediation roles of social engagement and cognitive function in sensitivity analysis 4 for CHARLS**

| **Dependent variable** | **Independent variable** | ***β*** | **Lower 95% CI** | **Upper 95% CI** | ***t*** | ***P*** |
| --- | --- | --- | --- | --- | --- | --- |
| M1 | X | -0.1518 | -0.279 | -0.0246 | -2.3402 | 0.0193 |
| M2 | X | -0.3484 | -0.5752 | -0.1217 | -3.0122 | 0.0026 |
| Y | X | 0.3627 | 0.0547 | 0.6708 | 2.3084 | 0.021 |
| Y | M1 | -0.1455 | -0.2127 | -0.0783 | -4.247 | < 0.001 |
| Y | M2 | -0.1245 | -0.1621 | -0.0868 | -6.4772 | < 0.001 |

*Notes*: *CI* confidence interval

*X* dual sensory impairment, *M1* social engagement, *M2* cognitive function, *Y* depressive symptoms

**Table S25. Bootstrap analysis of the mediating model in sensitivity analysis 4 for CHARLS**

| **Path** | **Effect** | **SE** | **95% CI** | | **Mediated proportion (%)** |
| --- | --- | --- | --- | --- | --- |
|  |  |  | **Lower** | **Upper** |  |
| Total effect | 0.428 | 0.1579 | 0.1185 | 0.7378 | - |
| Direct effect | 0.363 | 0.1571 | 0.0547 | 0.6708 | 85 |
| Total indirect effects | 0.066 | 0.0198 | 0.0278 | 0.1044 | 15 |
| Ind1, X→M1→Y | 0.022 | 0.0106 | 0.0036 | 0.0447 | 5 |
| Ind2, X→M2→Y | 0.043 | 0.0159 | 0.014 | 0.0763 | 10 |
| Ind1 - ind2 | -0.021 | 0.0184 | -0.0581 | 0.014 | - |

*Notes*: *SE* standard error, *CI* confidence interval

*X* dual sensory impairment, *M1* social engagement, *M2* cognitive function, *Y* depressive symptoms

**Identification of studies via databases**

Duplicate records removed

(n = 1112)

Records identified from database (n = 4175):

Pubmed (n = 1281)

Embase (n = 628)

Web of Science (n = 2022)

Medline (n = 244)

**Identification**

Records excluded (n = 3020)

Titles and abstracts screened

(n = 3063)

**Included**

**Screening**

Reports excluded (n = 35):

Study design is ineligible

(n = 17)

Exposure factors are ineligible (n = 8)

Outcomes are ineligible (n = 4)

Data are unavailable (n = 6)

Reports assessed for eligibility (n = 43)

Studies included in meta-analysis (n = 8)

**Figure S5. The PRISMA flow diagram**

**Table S26. Characteristics of the included studies**

| **Author, year** | **Country** | **Study Population** | **Sample size** | **Age (y)** | **Women (%)** | **Follow-up (y)** | **Assessment of DSI** | **Assessment of depressive symptoms** | **Covariates** |
| --- | --- | --- | --- | --- | --- | --- | --- | --- | --- |
| Chou et al., 2008 | China | England | 3782 | ≥ 65 | NR | 2 | Self-reported | CESD-8 | Age, gender, marital status, education, medical conditions in eight areas, functional health, smoking, frequency of alcohol consumption, emotional support, and negative interaction |
| Xie et al., 2021 | China | China | 2703 | ≥ 60 | NR | 4 | Self-reported | CESD-10 | Age, gender, education, marital status, perceived health condition, monthly household expenditure, cognitive ability, alcohol consumption, smoking status, and social activity |
| [Killeen](https://pubmed.ncbi.nlm.nih.gov/?size=50&term=Killeen+OJ&cauthor_id=35153667) et al., 2022 | the United States | the United States | 6253 | 65-74 | 56.5 | 8 | Self-reported | PHQ-2 | Sex, race/ethnicity, highest education, age, dementia status, number of chronic medical conditions, medicaid eligibility, proxy respondent, and survey round |
| Liu WW et al., 2022 | China | China | 8130 | 58.7 ± 9.4 | 51.99 | 4 | Self-reported | CESD-10 | Age, sex, education, area of residence, marital status, household income, smoking, drinking, [body mass index](https://www.sciencedirect.com/topics/medicine-and-dentistry/body-mass-index), hypertension, diabetes, and cardiovascular disease |
| Liu YG et al., 2022 | China | China | 9492 | 58.12 ± 9.00 | 48.06 | 7 | Self-reported | CESD-10 | Age, sex, residence, marital status, educational level, smoking history, alcohol consumption, hypertension, diabetes, heart disease, digestive disease, arthritis, wearing glasses, and hearing aids |
| [Ahrenfeldt](https://pubmed.ncbi.nlm.nih.gov/?size=50&term=Ahrenfeldt+LJ&cauthor_id=38695383) et al., 2024 | Denmark | Europe | 56847 | 63.2 ± 9.3 | 50.76 | ≥2 | Self-reported | EURO-D | Age, wave, region, partner in household, education, household income, and co-morbidities |
| Huang et al., 2024 | China | China | 4419 | 66.94 ± 7.49 | 83.0 | 3 | Self-reported | CESD-10 | Sex, educational level, residence, marital status, smoking, drinking, and heart disease |
| Liu et al., 2025 | China | China | 9780 | 58 (50, 64) | 49.42 | 7 | Self-reported | CESD-10 | Age, gender, education level, marital status, residence, sleep, social participation, cognitive ability, activities of daily living, hospitalization status, number of chronic diseases, and pain status |
| Ni et al., (current) | China | China | 5076 | 60.2 ± 6.9 | 54.3 | 7 | Self-reported | CESD-10 | Age, gender, education, marital status, residence/ethnicity, current smoking and drinking status, chronic diseases, and activities of daily living |
|  |  | the United States | 9169 | 65.6 ± 9.3 | 57.7 | 6 | Self-reported | CESD-8 |  |
|  |  | England | 5071 | 65.4 ± 8.3 | 54.1 | 6 | Self-reported | CESD-8 |  |

*Notes: DSI* dual sensory impairment, *NR* not reported, *CESD* Center for Epidemiologic Studies Depression Scale, *PHQ* Patient Health Questionnaire for Depression, *EURO-D* European Depression Scale.

**Table S27. The** **Newcastle-Ottawa Scale assessment**

| **Author, year** | **Selection** | | | |  | **Comparability** | |  | **Outcome** | | | **Total Score** |
| --- | --- | --- | --- | --- | --- | --- | --- | --- | --- | --- | --- | --- |
|  | **Representativeness of the exposed cohort** | **Selection of the non-exposed cohort** | **Ascertainment of exposure** | **Outcome of interest not present at start of the study** |  | **Control for primary confounders** | **Control for secondary confounders** |  | **Assessment of outcome** | **Duration of follow-up** | **Adequacy of follow-up** |  |
| Chou et al., 2008 | 1 | 1 | 1 | 1 |  | 1 | 1 |  | 1 | 1 | 1 | 9 |
| Xie et al., 2021 | 1 | 1 | 1 | 0 |  | 1 | 1 |  | 1 | 1 | 0 | 7 |
| [Killeen](https://pubmed.ncbi.nlm.nih.gov/?size=50&term=Killeen+OJ&cauthor_id=35153667) et al., 2022 | 1 | 1 | 1 | 1 |  | 1 | 1 |  | 1 | 1 | 0 | 8 |
| Liu Wenwen et al., 2022 | 1 | 1 | 1 | 1 |  | 1 | 1 |  | 1 | 1 | 1 | 9 |
| Liu Yunguang et al., 2022 | 1 | 1 | 1 | 1 |  | 1 | 1 |  | 1 | 1 | 1 | 9 |
| [Ahrenfeldt](https://pubmed.ncbi.nlm.nih.gov/?size=50&term=Ahrenfeldt+LJ&cauthor_id=38695383) et al., 2024 | 1 | 1 | 1 | 1 |  | 1 | 1 |  | 1 | 1 | 0 | 8 |
| Huang et al., 2024 | 1 | 1 | 1 | 0 |  | 0 | 1 |  | 1 | 1 | 1 | 7 |
| Liu et al., 2025 | 1 | 1 | 1 | 1 |  | 1 | 1 |  | 1 | 1 | 1 | 9 |
| Ni et al., current | 1 | 1 | 1 | 1 |  | 1 | 1 |  | 1 | 1 | 0 | 8 |

(a)

(b)

**Figure S6. The sensitivity analysis. (a) OR subgroup; (b) HR subgroup**

**Table S28. The subgroup analysis**

| **Categories** | **No. of OR estimate** | ***I*^2^ (OR)** | **OR (95% CI)** | ***P* ^a^ (OR)** | **No. of HR estimate** | ***I*^2^ (HR)** | **HR (95% CI)** | ***P* ^a^ (HR)** |
| --- | --- | --- | --- | --- | --- | --- | --- | --- |
| Region |  |  |  | 0.447 |  |  |  | 0.043 |
| China | 3 | 91.1% | 1.63 [1.12, 2.37] |  | 3 | 0 | 1.27 [1.18, 1.36] |  |
| Others | 4 | 10.3% | 1.90 [1.71, 2.11] |  | 1 | 0 | 1.67 [1.29, 2.16] |  |
| Sample size |  |  |  | 0.846 |  |  |  | / |
| <5000 | 3 | 47.1% | 1.79 [1.42, 2.25] |  | 0 | / | / |  |
| ≥5000 | 4 | 91.6% | 1.72 [1.28, 2.31] |  | 4 | 27.5% | 1.30 [1.19, 1.42] |  |
| Follow-up duration |  |  |  | 0.516 |  |  |  | 0.679 |
| <5 years | 4 | 22.9% | 1.89 [1.69, 2.12] |  | 1 | 0 | 1.27 [1.08, 1.49] |  |
| ≥5 years | 3 | 88.0% | 1.66 [1.12, 2.44] |  | 3 | 51.1% | 1.33 [1.17, 1.50] |  |
| NOS score |  |  |  | 0.203 |  |  |  | 0.043 |
| ≤8 | 6 | 87.5% | 1.78 [1.44, 2.20] |  | 1 | 0 | 1.67 [1.29, 2.16] |  |
| 9 | 1 | 0 | 1.31 [0.86, 2.00] |  | 3 | 0 | 1.27 [1.18, 1.36] |  |

*Notes*: *RR* Risk ratio, *CI* Confidence interval, *NOS* The Newcastle-Ottawa Scale.

a: *P*-value for group differences.
